# Supplementary material for: COPI-dependent intra-Golgi recycling at an intermediate stage of cisternal maturation
Source: bioRxiv. 2025 Sep 21:2025.09.20.677526. Preprint. [Version 1] doi: 10.1101/2025.09.20.677526 (PMC12458367; doi:10.1101/2025.09.20.677526)
Supplement: 1 [file NIHPP2025.09.20.677526V1-supplement-1.pdf]

bioRxiv preprint doi: https://doi.org/10.1101/2025.09.20.677526; this version posted September 21, 2025. The copyright holder for this preprint (which was not certified by peer review) is the author/funder, who has granted bioRxiv a license to display the preprint in perpetuity. It is made available under aCC-BY 4.0 International license.

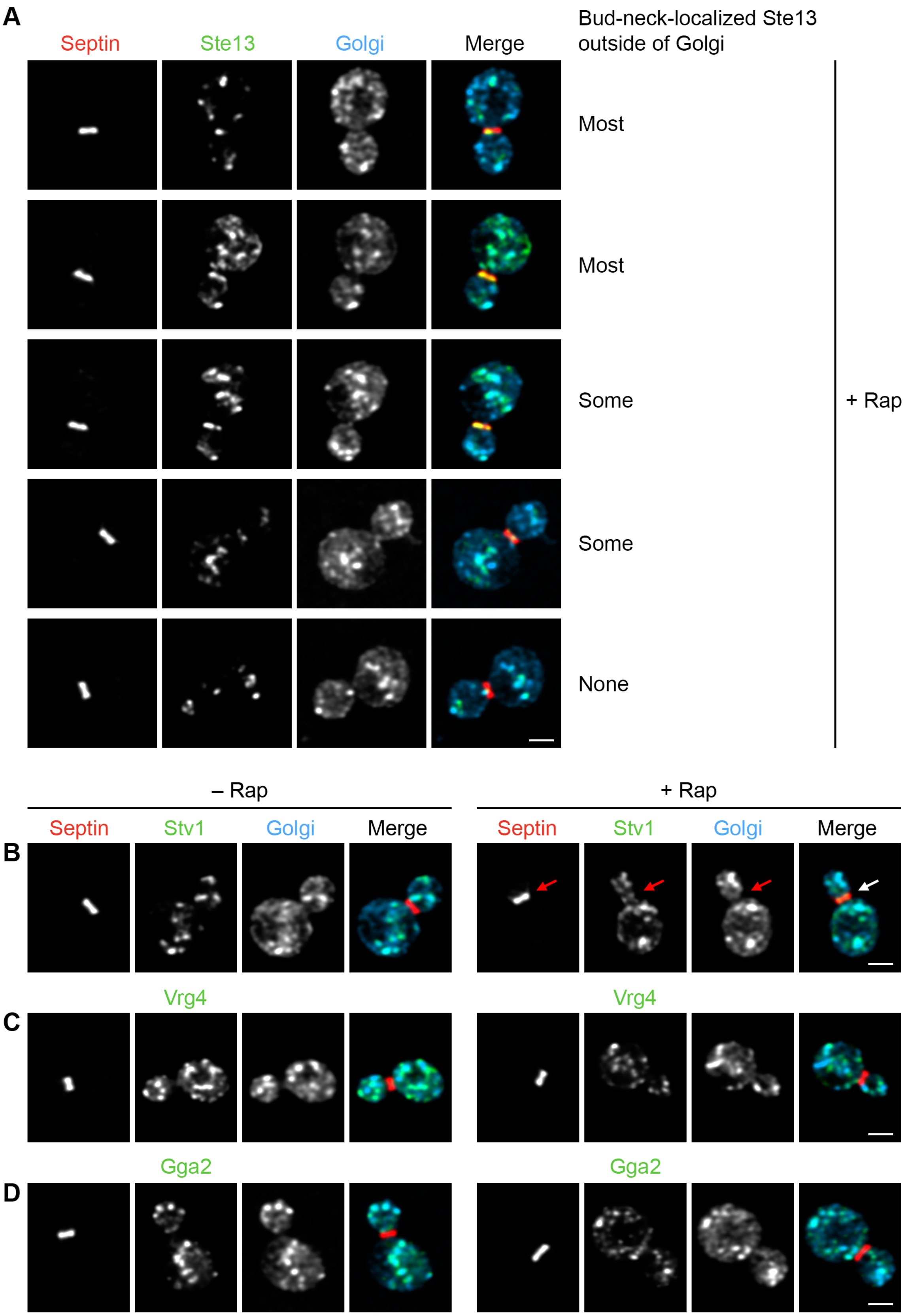

bioRxiv preprint doi: https://doi.org/10.1101/2025.09.20.677526; this version posted September 21, 2025. The copyright holder for this preprint (which was not certified by peer review) is the author/funder, who has granted bioRxiv a license to display the preprint in perpetuity. It is made available under aCC-BY 4.0 International license.

Figure S1. **Supplementary data for Fig. 2.** In each part of the figure, Golgi cisternae were marked by HaloTag-labeled Ric1 and Sec7 (blue), and the scale bar is 2 μm. **(A)** A panel of representative images showing capture with Kex2-FRB of GFP-tagged Ste13 (green) by an FKBP-tagged septin (red) after treatment for 5 min with rapamycin ("Rap"). The labels "Most", "Some", and "None" mark representative examples for the image categories used in Fig. 2 A. **(B)** Capture with Kex2-FRB of GFP-tagged Stv1 (green) by an FKBP-tagged septin (red) after treatment for 5 min with rapamycin. Arrows indicate non-Golgi signal at the bud neck. **(C)** No capture with Kex2-FRB of GFP-tagged Vrg4 (green) by an FKBP-tagged septin (red) after treatment for 5 min with rapamycin. **(D)** Minimal capture with Kex2-FRB of GFP-tagged Gga2 (green) by an FKBP-tagged septin (red) after treatment for 5 min with rapamycin.

bioRxiv preprint doi: https://doi.org/10.1101/2025.09.20.677526; this version posted September 21, 2025. The copyright holder for this preprint (which was not certified by peer review) is the author/funder, who has granted bioRxiv a license to display the preprint in perpetuity. It is made available under aCC-BY 4.0 International license.

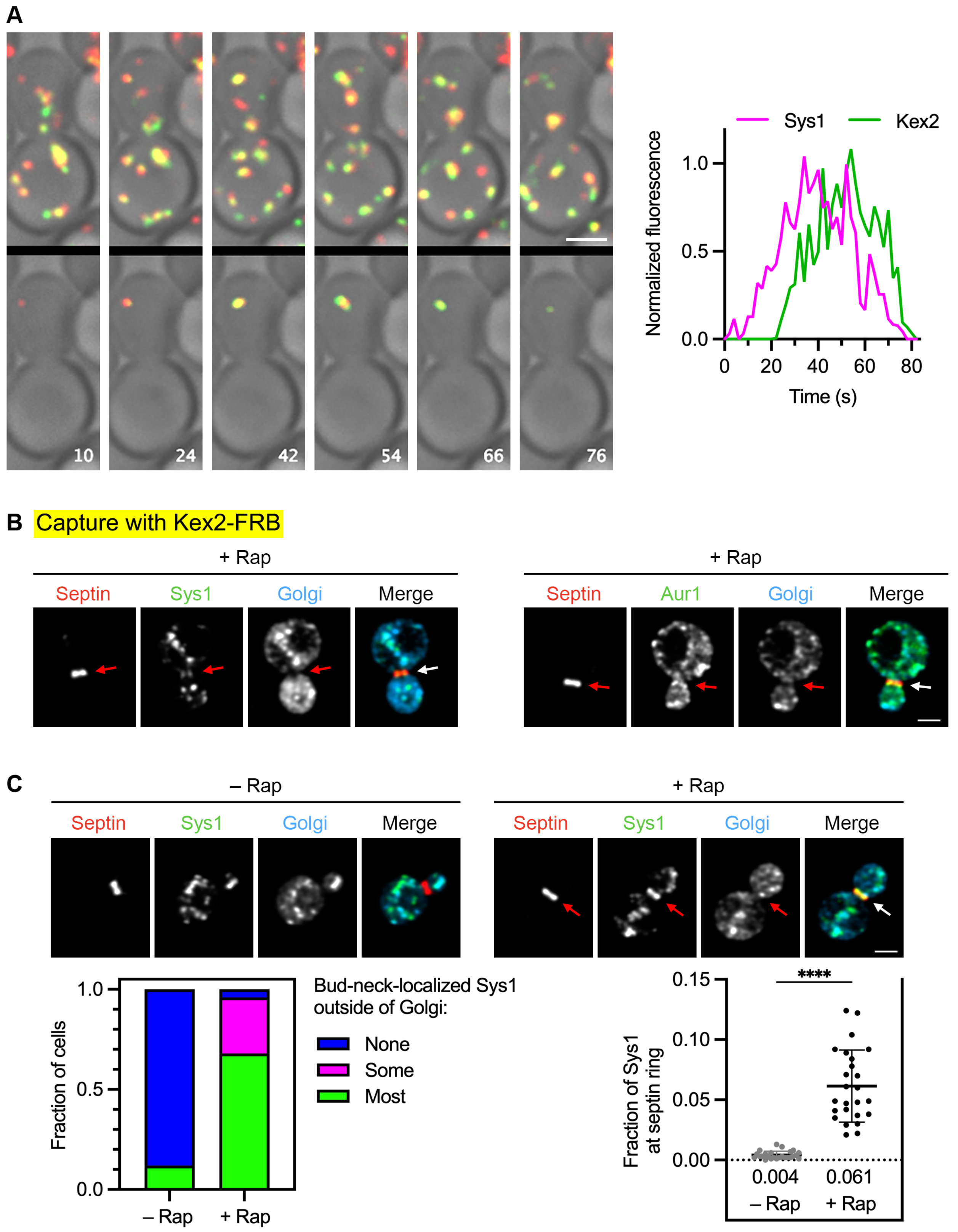

bioRxiv preprint doi: https://doi.org/10.1101/2025.09.20.677526; this version posted September 21, 2025. The copyright holder for this preprint (which was not certified by peer review) is the author/funder, who has granted bioRxiv a license to display the preprint in perpetuity. It is made available under aCC-BY 4.0 International license.

D

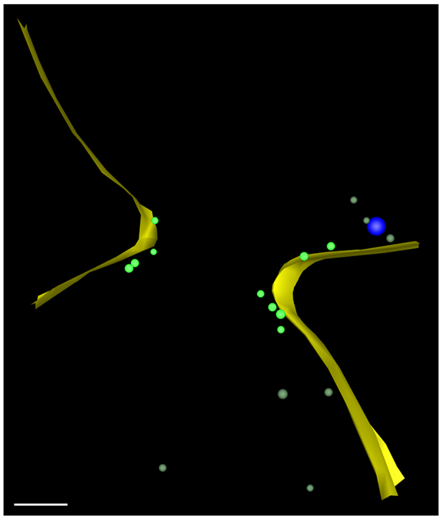

E

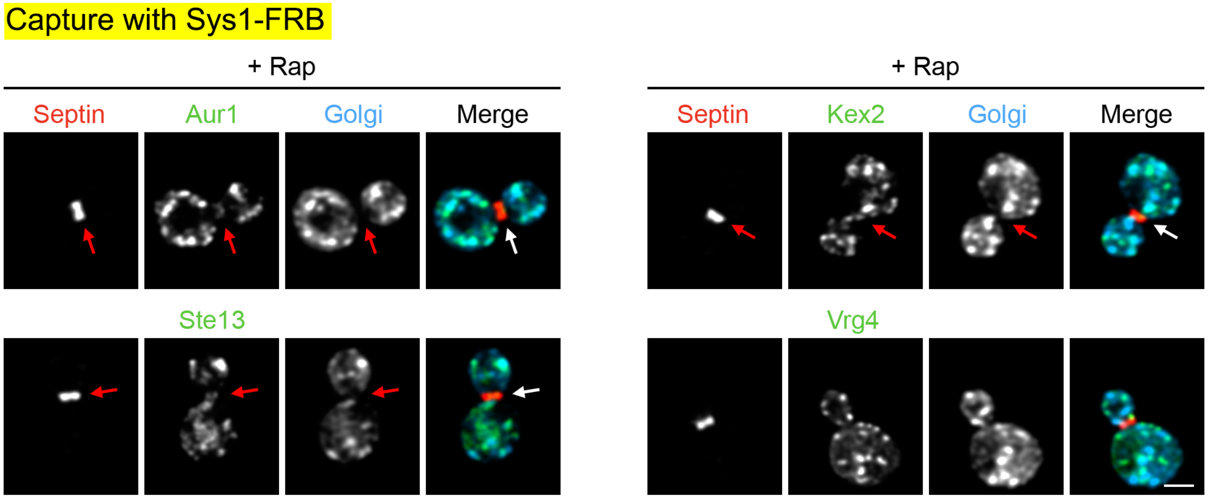

bioRxiv preprint doi: https://doi.org/10.1101/2025.09.20.677526; this version posted September 21, 2025. The copyright holder for this preprint (which was not certified by peer review) is the author/funder, who has granted bioRxiv a license to display the preprint in perpetuity. It is made available under aCC-BY 4.0 International license.

Figure S2. **Supplementary data for Fig. 3. (A)** Frames from a representative 4D confocal movie of Sys1-HaloTag (red) and Kex2-GFP (green), and kinetic traces from an individual cisterna in the movie. Depicted at the left are average projected z-stacks at the indicated time points from Video 2. The upper row shows the complete projections, and the lower row shows edited projections that include only the cisterna that was tracked. Scale bar, 2 µm. Plotted at the right are normalized fluorescence intensities for the cisterna tracked in the movie. **(B)** Representative images showing capture with Kex2-FRB of GFP-tagged Sys1 or Aur1 (green) by an FKBP-tagged septin (red) after treatment for 5 min with rapamycin. Arrows indicate non-Golgi signal at the bud neck. **(C)** Rapamycin-dependent capture by an FKBP-tagged septin (red) of Sys1-FRB-GFP (green). Arrows indicate non-Golgi signal at the bud neck after treatment for 5 min with rapamycin. At the lower left, capture of Sys1-FRB-GFP at the bud neck was quantified by assigning cells to categories as described in Fig. 1 C. At the lower right, capture of Sys1-FRB-GFP at the bud neck was quantified numerically as described in Fig. 1 C. ****, significant at P value <0.0001. **(D)** Cryo-ET of vesicles captured at the bud neck using Sys1-FRB. A log-phase culture of cells expressing Sys1-FRB and Shs1-FKBP was treated with rapamycin for 5 min followed by cryopreservation and processing for cryo-ET. Shown is the model of a SIRT-reconstructed tomogram from a large budded cell. The full data set is shown in Video 3. A vesicle was counted as putatively captured if its membrane was no more than 83 nm from a point on the plasma membrane within 200 nm from the center of the bud neck. For the 4 non-rapamycin-treated cells examined, the number of vesicles meeting this criterion ranged from 0 to 2 (mean = 1.0). For the 4 rapamycin-treated cells examined, the number of putatively captured vesicles ranged from 6 to 13 (mean = 10.0). Scale bar, 250 nm. See Fig. 1 D for further details. **(E)** Representative images showing capture with Sys1-FRB of GFP-tagged Aur1, Kex2, or Ste13 (green) by an FKBP-tagged septin (red), and minimal capture with Sys1-FRB of Vrg4 (green) by an FKBP-tagged septin (red), all after treatment for 5 min with rapamycin. Arrows indicate non-Golgi signal at the bud neck.

bioRxiv preprint doi: https://doi.org/10.1101/2025.09.20.677526; this version posted September 21, 2025. The copyright holder for this preprint (which was not certified by peer review) is the author/funder, who has granted bioRxiv a license to display the preprint in perpetuity. It is made available under aCC-BY 4.0 International license.

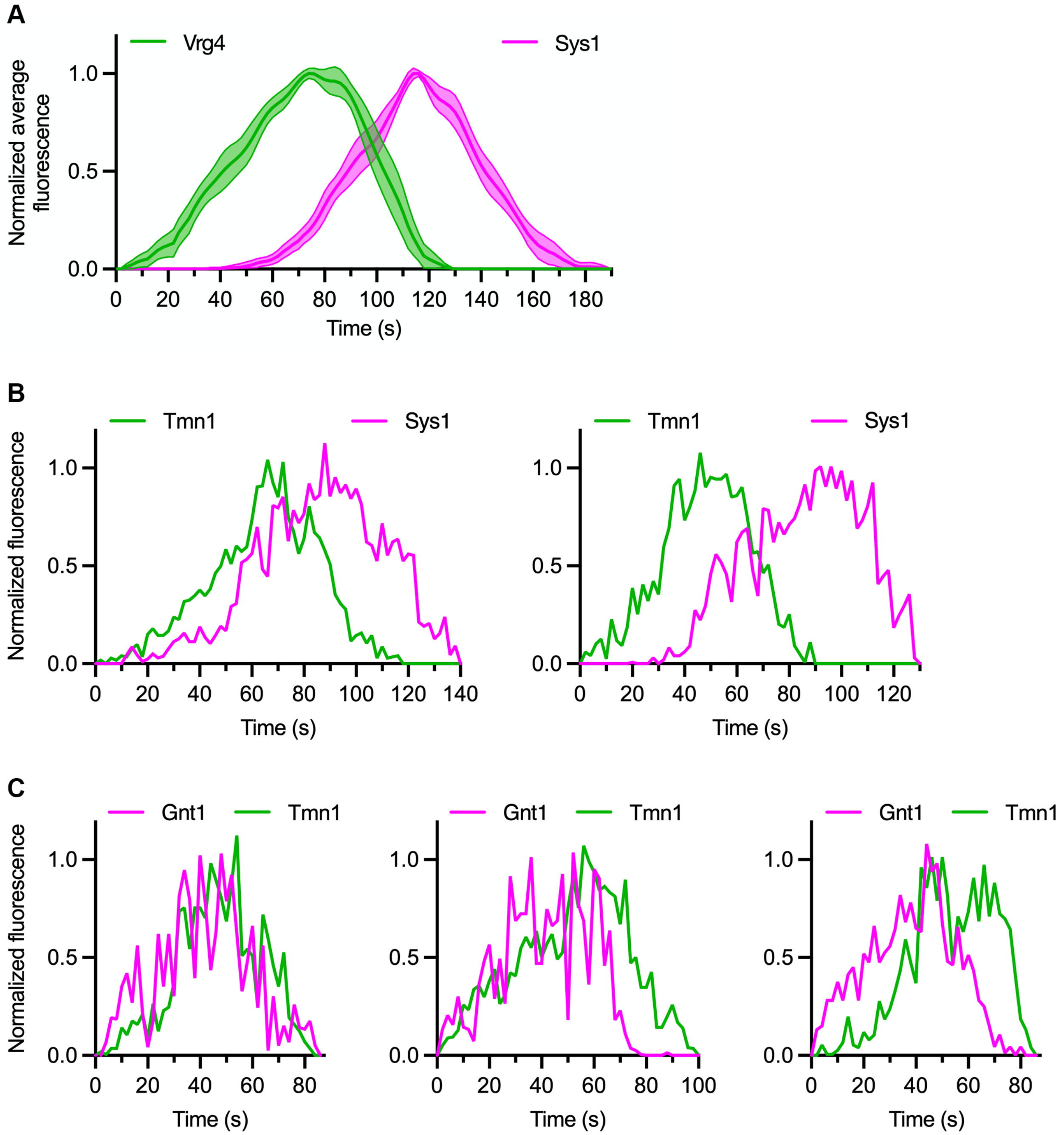

bioRxiv preprint doi: https://doi.org/10.1101/2025.09.20.677526; this version posted September 21, 2025. The copyright holder for this preprint (which was not certified by peer review) is the author/funder, who has granted bioRxiv a license to display the preprint in perpetuity. It is made available under aCC-BY 4.0 International license.

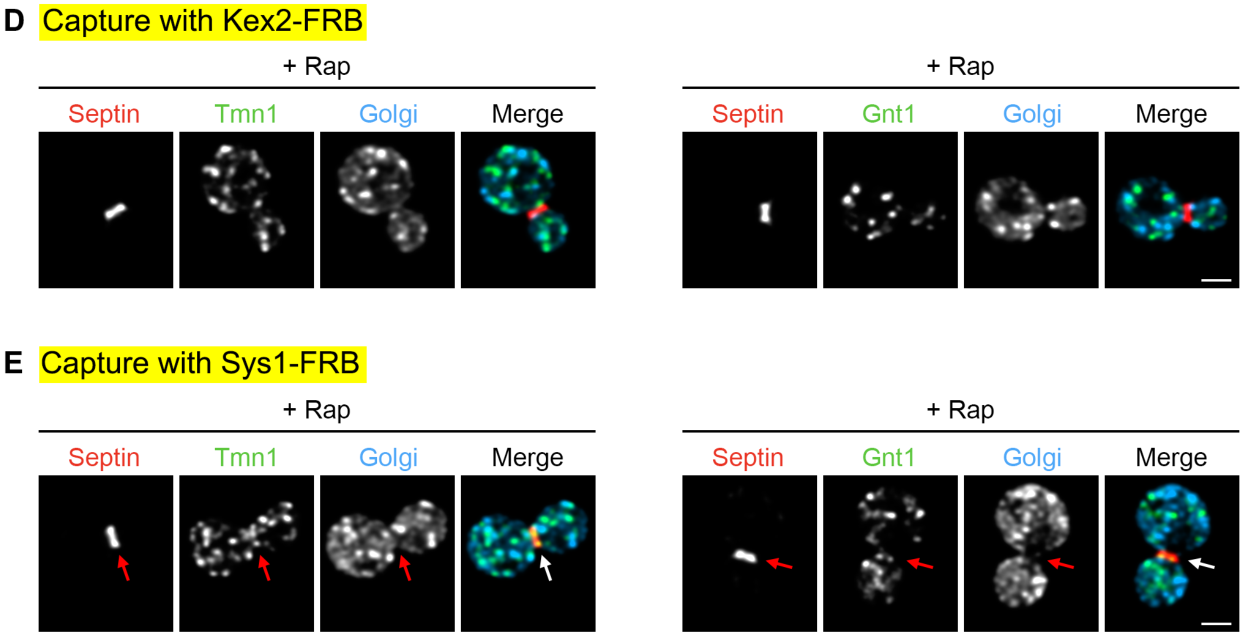

Figure S3. **Supplementary data for Fig. 4. (A)** Golgi maturation kinetics of GFP-tagged Vrg4 compared to HaloTag-labeled Sys1. Shown are normalized and averaged traces for 13 individual cisternae. **(B)** Kinetic traces for two representative cisternae illustrating variations in the relative arrival and departure times of GFP-Tmn1 versus Sys1-HaloTag. Plotted are normalized fluorescence intensities. **(C)** Kinetic traces for three representative cisternae illustrating variations in the relative arrival and departure times of Gnt1-HaloTag versus GFP-Tmn1. Plotted are normalized fluorescence intensities. **(D)** Representative images showing minimal capture with Kex2-FRB of GFP-tagged Tmn1 (green) or no capture with Kex2-FRB of GFP-tagged Gnt1 (green) by an FKBP-tagged septin (red) after treatment for 5 min with rapamycin. **(E)** Representative images showing capture with Sys1-FRB of GFP-tagged Tmn1 or Gnt1 (green) by an FKBP-tagged septin (red) after treatment for 5 min with rapamycin. Arrows indicate non-Golgi signal at the bud neck.

bioRxiv preprint doi: https://doi.org/10.1101/2025.09.20.677526; this version posted September 21, 2025. The copyright holder for this preprint (which was not certified by peer review) is the author/funder, who has granted bioRxiv a license to display the preprint in perpetuity. It is made available under aCC-BY 4.0 International license.

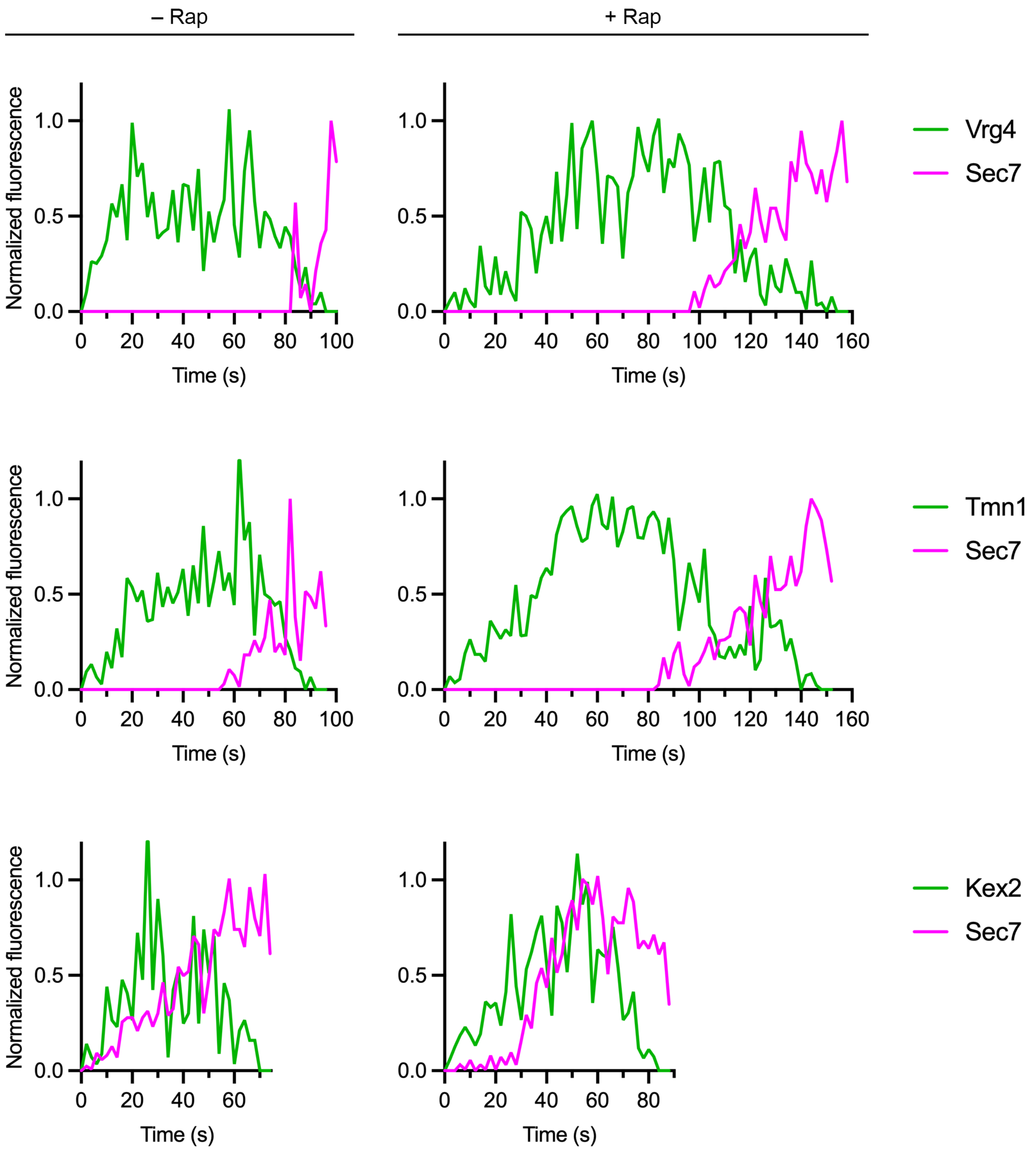

Figure S4. **Supplementary data for Fig. 6.** Kinetic traces for representative cisternae illustrate the residence times of GFP-tagged Vrg4, Tmn1, and Kex2 in Golgi cisternae either with normal COPI activity ("– Rap") or with reduced COPI activity caused by brief rapamycin treatment ("+ Rap"). The analysis included partial companion traces of the late Golgi marker Sec7-mScarlet. Plotted are normalized fluorescence intensities.

bioRxiv preprint doi: https://doi.org/10.1101/2025.09.20.677526; this version posted September 21, 2025. The copyright holder for this preprint (which was not certified by peer review) is the author/funder, who has granted bioRxiv a license to display the preprint in perpetuity. It is made available under aCC-BY 4.0 International license.

A

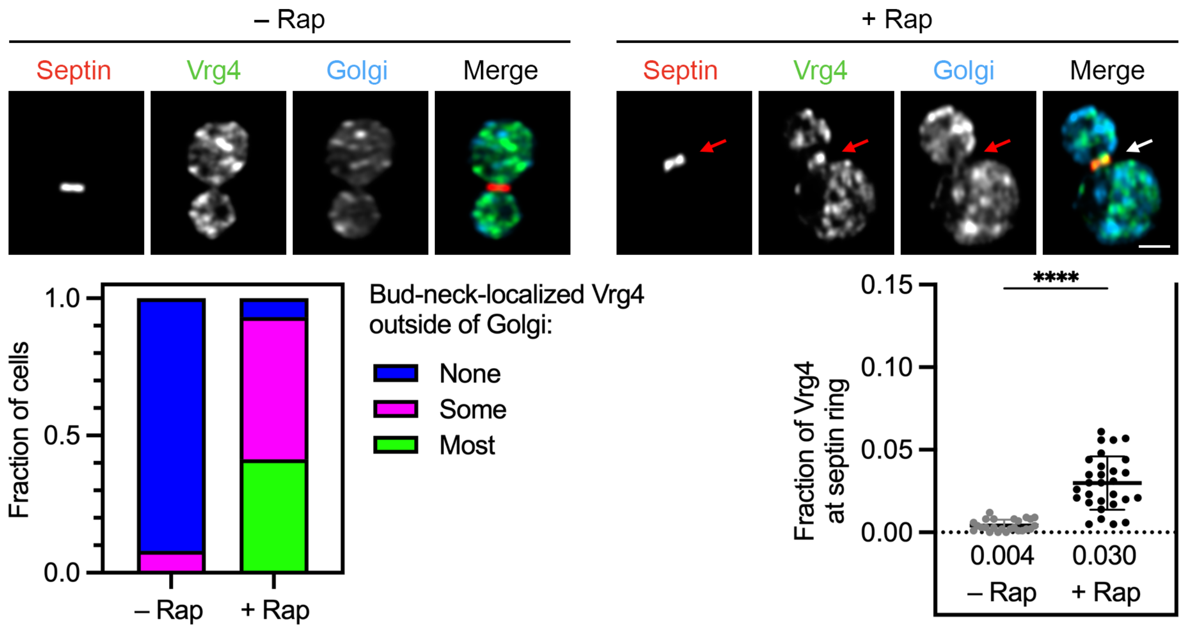

B

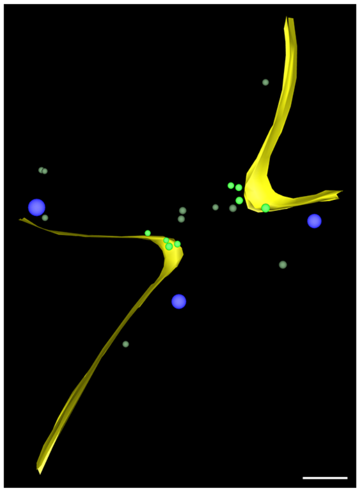

bioRxiv preprint doi: https://doi.org/10.1101/2025.09.20.677526; this version posted September 21, 2025. The copyright holder for this preprint (which was not certified by peer review) is the author/funder, who has granted bioRxiv a license to display the preprint in perpetuity. It is made available under aCC-BY 4.0 International license.

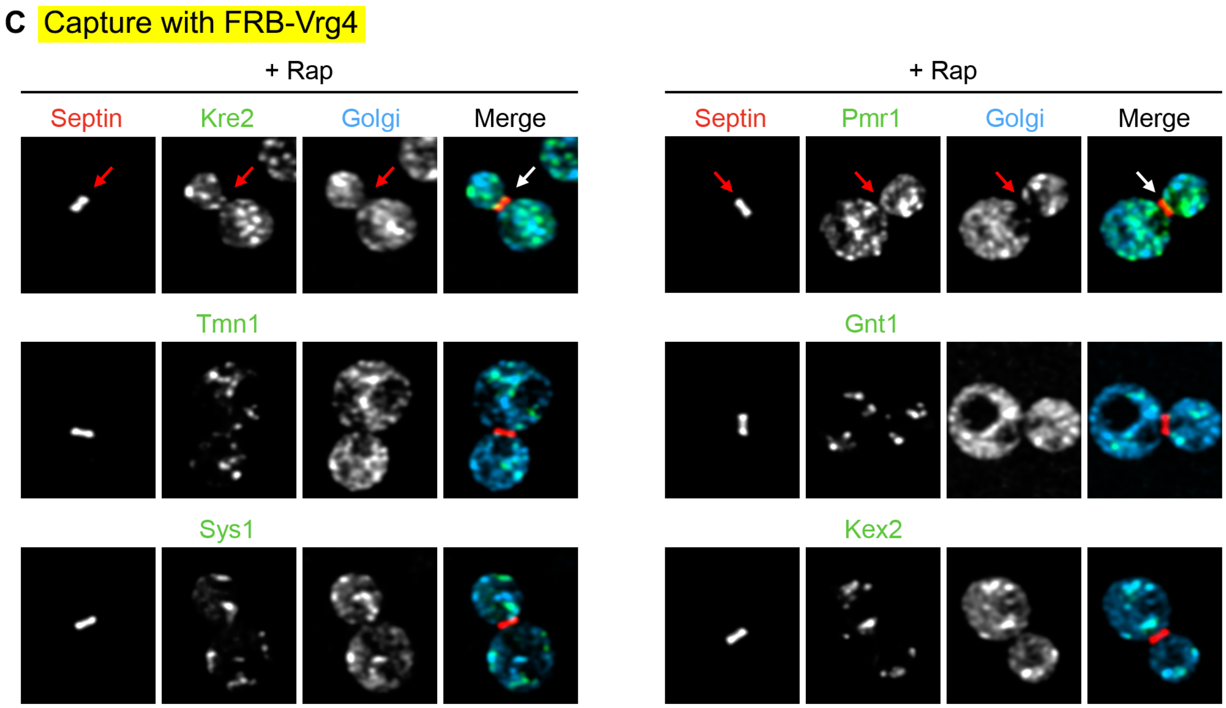

Figure S5. **Supplementary data for Fig. 7. (A)** Rapamycin-dependent capture by an FKBP-tagged septin (red) of FRB-GFP-Vrg4 (green). Arrows indicate non-Golgi signal at the bud neck after treatment for 5 min with rapamycin (“Rap”). At the lower left, capture of FRB-GFP-Vrg4 at the bud neck was quantified by assigning cells to categories as described in Fig. 1 C. At the lower right, capture of FRB-GFP-Vrg4 at the bud neck was quantified numerically as described in Fig. 1 C. ****, significant at P value <0.0001. **(B)** Cryo-ET of vesicles captured at the bud neck using FRB-Vrg4. A log-phase culture of cells expressing FRB-Vrg4 and Shs1-FKBP was treated with rapamycin for 5 min followed by cryopreservation and processing for cryo-ET. Shown is the model of a SIRT-reconstructed tomogram from a large budded cell. The full data set is shown in Video 6. A vesicle was counted as putatively captured if its membrane was no more than 66 nm from a point on the plasma membrane within 200 nm from the center of the bud neck. For the 6 non-rapamycin-treated cells examined, the number of vesicles meeting this criterion ranged from 0 to 2 (mean = 0.8). For the 6 rapamycin-treated cells examined, the number of putatively captured vesicles ranged from 2 to 7 (mean = 5.0). Scale bar, 250 nm. See Fig. 1 D for further details. **(C)** Representative images showing capture with FRB-Vrg4 of GFP-tagged Kre2 and Pmr1 (green) by an FKBP-tagged septin (red), minimal capture with FRB-Vrg4 of GFP-tagged Tmn1 and Sys1 (green) by an FKBP-tagged septin (red), and no capture with FRB-Vrg4 of GFP-tagged Gnt1 or Kex2 (green) by an FKBP-tagged septin (red), all after treatment for 5 min with rapamycin. Arrows indicate non-Golgi signal at the bud neck.

bioRxiv preprint doi: https://doi.org/10.1101/2025.09.20.677526; this version posted September 21, 2025. The copyright holder for this preprint (which was not certified by peer review) is the author/funder, who has granted bioRxiv a license to display the preprint in perpetuity. It is made available under aCC-BY 4.0 International license.

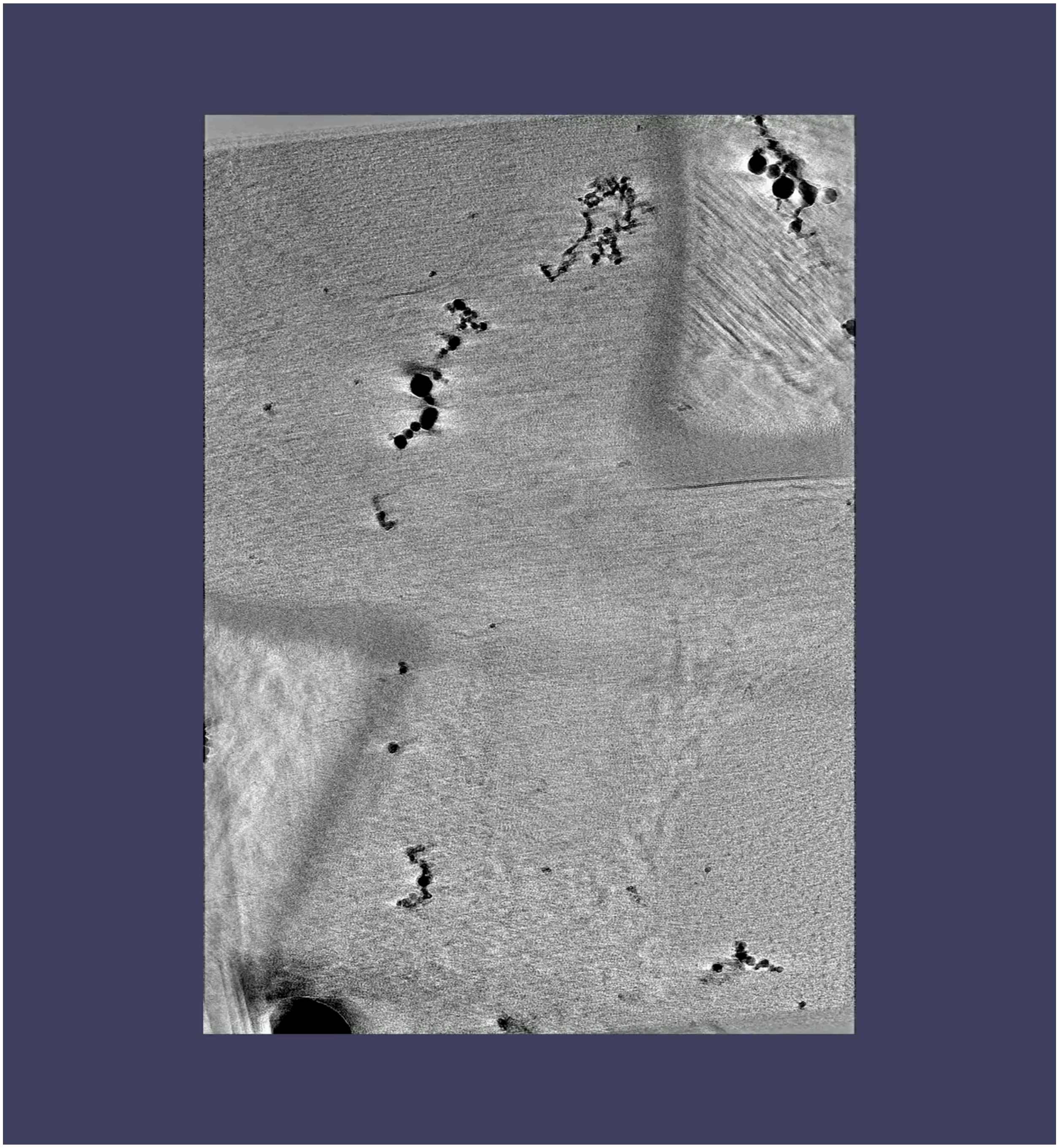

bioRxiv preprint doi: https://doi.org/10.1101/2025.09.20.677526; this version posted September 21, 2025. The copyright holder for this preprint (which was not certified by peer review) is the author/funder, who has granted bioRxiv a license to display the preprint in perpetuity. It is made available under aCC-BY 4.0 International license.

Video 1. **Tomographic sections and modeling of part of the bud neck region in a cell with Kex2-FRB-containing vesicles captured by an FKBP-tagged septin.** A log-phase culture of cells expressing Kex2-FRB and Shs1-FKBP was treated for 5 min with rapamycin prior to cryopreservation and processing for cryo-ET. The first third of the video shows every fifth section of the SIRT-reconstructed tomogram. The second third of the video shows the same tomographic sections after contours were segmented to mark the cell cortex (yellow), a secretory vesicle (blue), putatively captured non-secretory vesicles (bright green), and other non-secretory vesicles (dull green). Also marked is a mitochondrion (cyan). The final third of the video shows a rotation of the tomographic model.

bioRxiv preprint doi: https://doi.org/10.1101/2025.09.20.677526; this version posted September 21, 2025. The copyright holder for this preprint (which was not certified by peer review) is the author/funder, who has granted bioRxiv a license to display the preprint in perpetuity. It is made available under aCC-BY 4.0 International license.

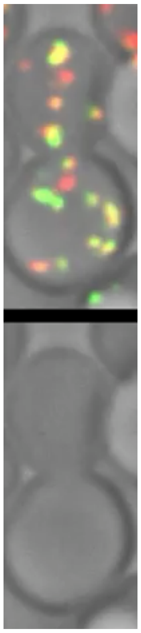

Video 2. **Representative 4D confocal movie of Sys1-HaloTag and Kex2-GFP.** 3D z-stacks for the individual time points were average projected. The upper row shows the complete projections, and the lower row shows edited projections that include only the cisterna that was tracked. Intervals between frames are 2 s. The overlaid numbers in a subset of the frames represent the time in seconds after the cisterna that was tracked first became detectable. See Fig. S2 A for further details.

bioRxiv preprint doi: https://doi.org/10.1101/2025.09.20.677526; this version posted September 21, 2025. The copyright holder for this preprint (which was not certified by peer review) is the author/funder, who has granted bioRxiv a license to display the preprint in perpetuity. It is made available under aCC-BY 4.0 International license.

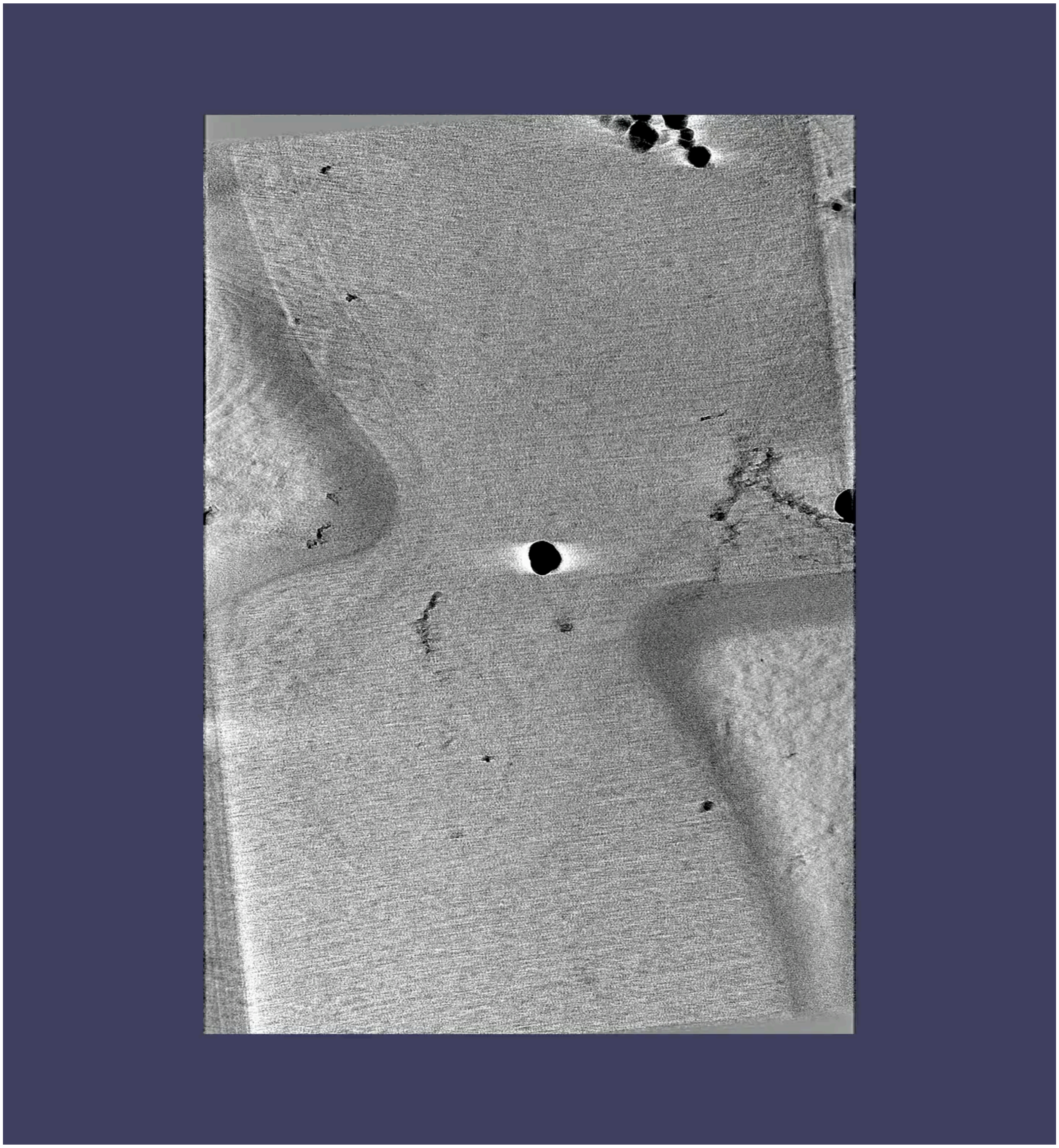

Video 3. **Tomographic sections and modeling of part of the bud neck region in a cell with Sys1-FRB-containing vesicles captured by an FKBP-tagged septin.** A log-phase culture of cells expressing Sys1-FRB and Shs1-FKBP was treated for 5 min with rapamycin prior to cryopreservation and processing for cryo-ET. Further details are as in Video 1, except that the nuclear envelope is marked in magenta.

bioRxiv preprint doi: https://doi.org/10.1101/2025.09.20.677526; this version posted September 21, 2025. The copyright holder for this preprint (which was not certified by peer review) is the author/funder, who has granted bioRxiv a license to display the preprint in perpetuity. It is made available under aCC-BY 4.0 International license.

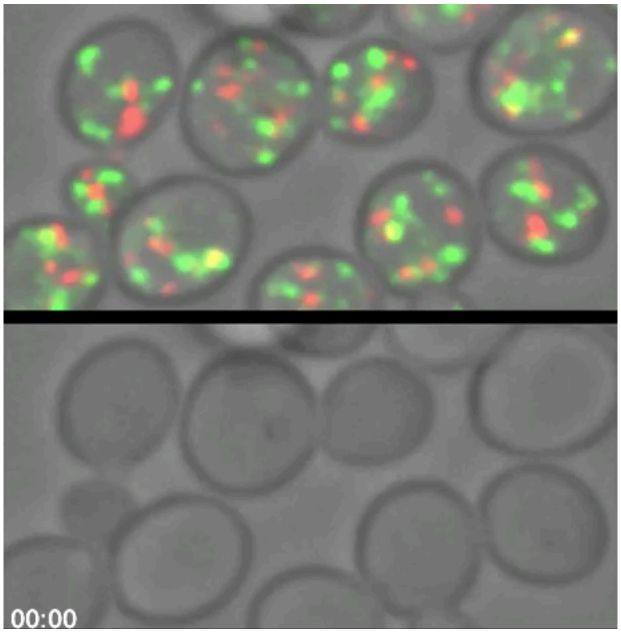

Video 4. **Representative 4D confocal movie of Vrg4 dynamics in Golgi cisternae with normal COPI activity.** Intervals between frames are 2 s. This movie corresponds to the "–Rap" trace at the top of Fig. S4, with time zero in that trace corresponding to 2:22 for the numbering shown here. 3D z-stacks for the individual time points were average projected. The upper row shows the complete projections, and the lower row shows edited projections that include only the single cisterna that was tracked. Vrg4 is green and Sec7 is red.

bioRxiv preprint doi: https://doi.org/10.1101/2025.09.20.677526; this version posted September 21, 2025. The copyright holder for this preprint (which was not certified by peer review) is the author/funder, who has granted bioRxiv a license to display the preprint in perpetuity. It is made available under aCC-BY 4.0 International license.

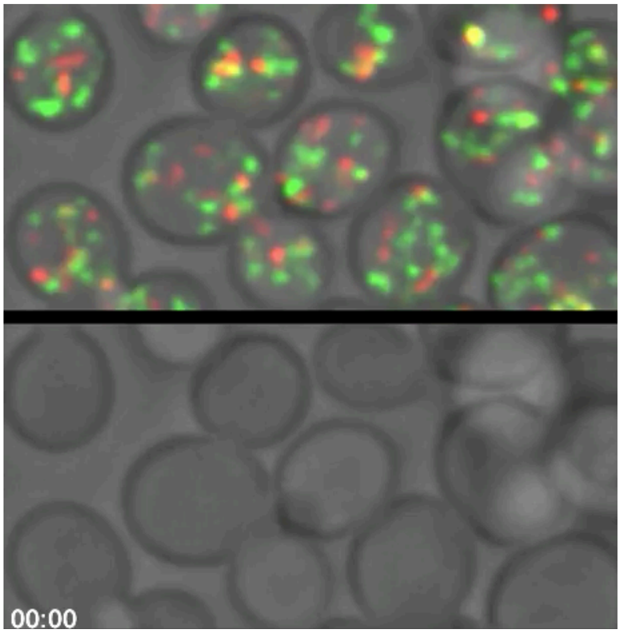

Video 5. **Representative 4D confocal movie of Vrg4 dynamics in Golgi cisternae with compromised COPI activity.** Time zero in this movie is 4 min after rapamycin addition, and intervals between frames are 2 s. This movie corresponds to the "+ Rap" trace at the top of Fig. S4, with time zero in that trace corresponding to 2:56 for the numbering shown here. 3D z-stacks for the individual time points were average projected. The upper row shows the complete projections, and the lower row shows edited projections that include only the single cisterna that was tracked. Vrg4 is green and Sec7 is red.

bioRxiv preprint doi: https://doi.org/10.1101/2025.09.20.677526; this version posted September 21, 2025. The copyright holder for this preprint (which was not certified by peer review) is the author/funder, who has granted bioRxiv a license to display the preprint in perpetuity. It is made available under aCC-BY 4.0 International license.

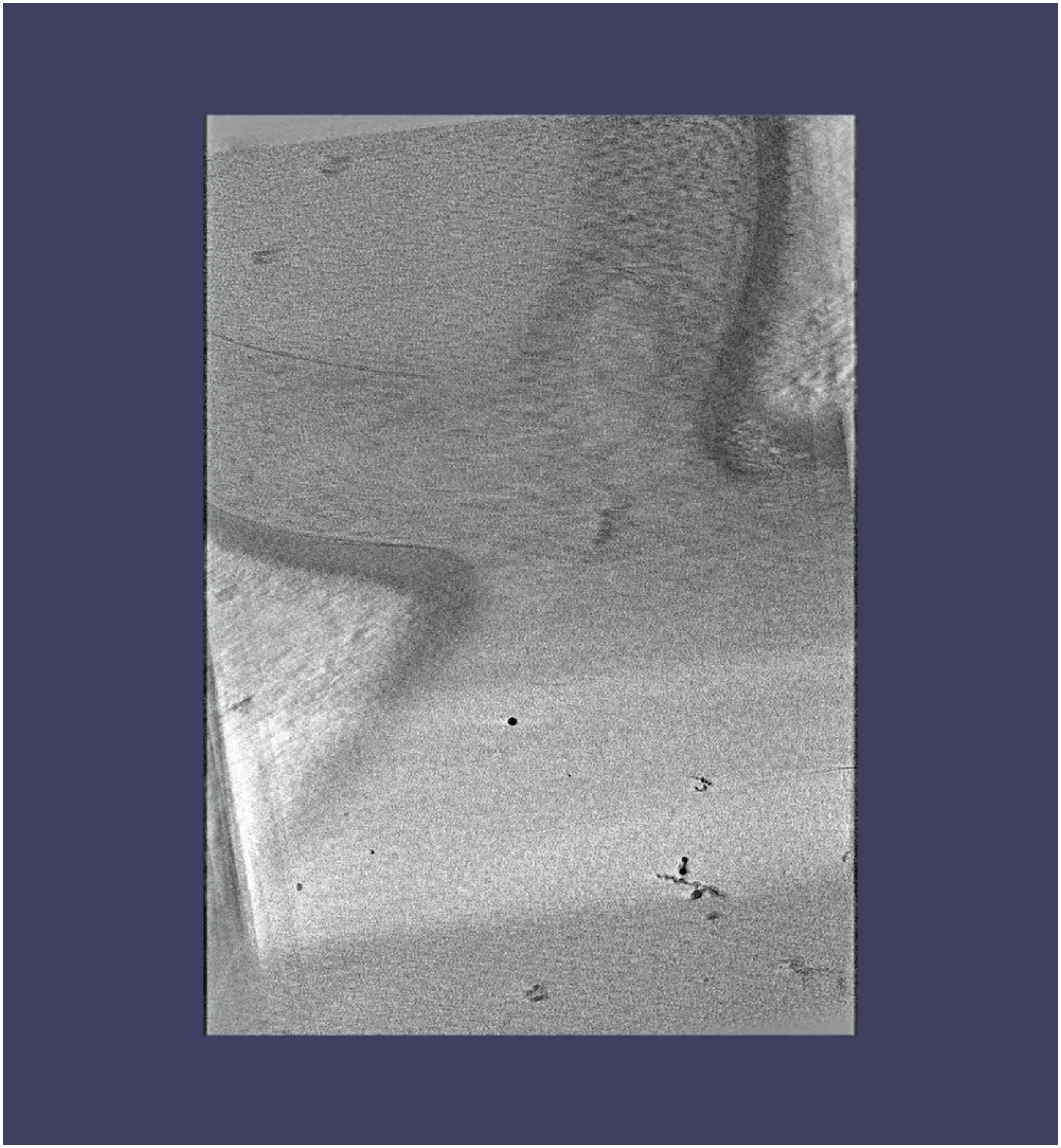

Video 6. **Tomographic sections and modeling of part of the bud neck region in a cell with FRB-Vrg4-containing vesicles captured by an FKBP-tagged septin.** A log-phase culture of cells expressing FRB-Vrg4 and Shs1-FKBP was treated for 5 min with rapamycin prior to cryopreservation and processing for cryo-ET. Further details are as in Video 1.
